# Photoacoustic viscoelasticity assessment of prefrontal cortex and cerebellum in normal and prenatal valproic acid-exposed rats

**DOI:** 10.1016/j.pacs.2024.100590

**Published:** 2024-01-20

**Authors:** Zahra Hosseindokht, Shima Davoudi, Mona Rahdar, Mahyar Janahmadi, Mohammadreza Kolahdouz, Pezhman Sasanpoour

**Affiliations:** a School of Electrical and Computer Engineering, College of Engineering, University of Tehran, Tehran, Iran; b Department of Physiology, School of Medicine, Shahid Beheshti University of Medical Sciences, Tehran, Iran; c Department of Medical Physics and Biomedical Engineering, School of Medicine, Shahid Beheshti University of Medical Sciences, Tehran, Iran

**Keywords:** Photoacoustic, Brain Tissue, Viscoelasticity, Autism, Photoacoustic Viscoelasticity, Valproic acid

## Abstract

Mechanical properties of brain tissues are from principal features from different points of view; diagnosis, the performance of the brain and neurological disorders. Particularly viscoelastic properties of the brain tissues are determinative. In this study based on a proposed accurate and non-invasive method, we have measured the viscoelastic properties of prefrontal cortex and cerebellum, two important brain regions involved in motor learning and pathophysiology of autism spectrum disorder (ASD). In this regard, using photoacoustic systems, viscoelastic properties of tissues from the cerebellum and prefrontal cortex of normal and prenatal VPA (Valproic acid)-exposed (i.e. autistic-like) offspring rats are measured. Results of our study show that the cerebellums of normal tissues are stiffer than the tissue obtained from autistic-like rats, while the viscoelasticity of the prefrontal cortex of normal tissues is higher than that of autistic ones. The proposed method for the measurement of viscoelastic properties of the brain tissue has the potential not only for the fundamental studies but as a diagnosis technique.

## Graphical Abstract

**Figure:**
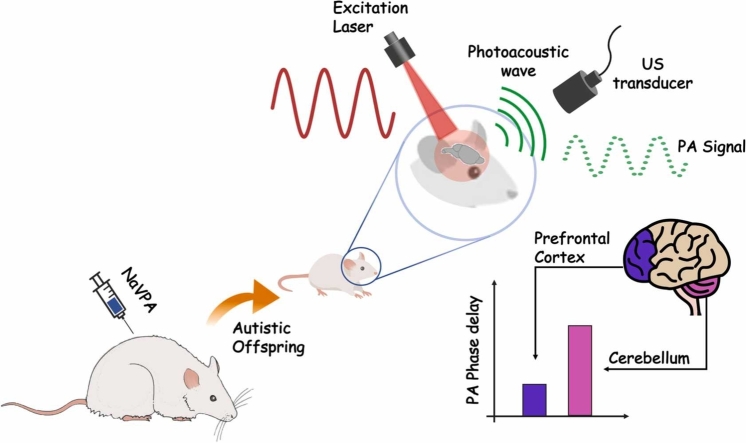

## 1 Introduction

Autism spectrum disorder (ASD) is a group of the most frequently diagnosed neurodevelopmental disabilities characterized by impairments in social skills, a range of deficits in cognitive function, and altered motor learning [1], [2]. The prevalence of ASD has been reported as 1–1.7%, with an increasing trend over time [3]. Although the etiology of ASD is poorly understood, and it makes diagnosis and treatment challenging, many studies have focused on the genetic and epigenetic factors contributing to autism pathophysiology [4]. However, mechanical factors that may severely affect brain pathology have not yet been thoroughly assessed in ASD. Structural and mechanical properties of the brain are interlinked with brain composition [5] and gray and white matter properties [6]. Furthermore, it is generally agreed that the brain tissue’s mechanical properties not only strongly influence normal brain function and development but also can alter the progression of neurological disorders [5]. Several studies have shown a direct correlation between abnormal mechanical properties and neurodegenerative conditions, such as Alzheimer’s disease, encephalomyelitis, and multiple sclerosis [7]. Currently, some ASD-related quantitative differences in brain morphometry in various regions (particularly in the prefrontal cortex and cerebellum) have been rendered [5]. Abnormalities in white matter and disorganized neuronal connectivity have also been previously shown in ASD brains [7]. However, whether any mechanical changes can be detected in the brain areas and regions in an autistic-like model (such as maternal exposure of rats to VPA) has not been reported yet.

Examining the local mechanical properties of the brain tissue in pathological conditions would enable scientists to shed light on the new area of exploration for treatment targets or diagnostic markers in ASD that may affect brain structure. Moreover, the brain is mechanically more compliant than other biological tissues and can exhibit viscoelastic deformations [7]. A quantitative evaluation of the viscoelasticity characteristics of the multiple brain areas might pave an appealing new way for finding the underlying mechanisms contributing to the functioning of the normal brain and neurodevelopmental disorders. There are several studies assessing various mechanical properties of the brain tissue, including elasticity [8], [9] and viscoelasticity [6], [10] in different brain areas and under different conditions such as ageing. Although numerous studies have been conducted on the viscoelasticity of the brain tissue, there is still a lack of information regarding how brain diseases may affect these mechanical properties.

With the recent acknowledgement of the brain areas that are crucial for social and cognitive functions, the cerebellum and prefrontal cortex abnormalities are associated with autistic symptoms [11]. Accumulating evidence indicates that the cerebellum is involved not only in motor coordination but also in cognitive and social functions [12]. Structural and functional cerebellar abnormalities have been frequently reported in patients diagnosed with autism [11], [12], [13]. Recent studies have suggested a pivotal implication of the prefrontal cortex in autism to explain some symptoms [14], [15]. The prefrontal cortex is crucial for social interaction, emotional behaviors and higher-order cognitive, language, and executive functions that are disrupted in ASD [16]. Studies investigating structurally and functionality in ASD patients have shown a positive and direct correlation between prefrontal cortex abnormalities and autism traits [11]. In addition, cerebellar-prefrontal cortex functional connectivity changes have also been identified in human and mouse models of ASD [17]. In the present study, we attempted to examine whether induction of autism may affect the viscoelastic properties of these brain tissue. Mechanical properties of the brain tissue play an important role in modulating the function and dysfunction of the brain [18]; therefore, characterizing the mechanical properties of the brain tissue may help better understand the pathological changes that occur in brain diseases.

There have been several methods for studying the viscoelasticity of brain tissue such as rheology in which externally controlled deformation is applied to the brain sample and the resulting strain and stress are measured [19], [20]; ultrasound elastography (USE) which is composed of an acoustic actuation for disturbance introduction and ultrasound imaging followed by fitting to a rheological model [21]; indentation which involves the employment of a small force to the surface of brain tissue following by measuring its resulting deformation [6], [22]; magnetic resonance elastography (MRE) which uses magnetic resonance imaging (MRI) to capture images of the tissue and calculate the displacement caused by an external vibration [23], [24]; and on the smaller scale, atomic force microscopy (AFM) which has been used for evaluating the mechanical properties of biological samples including cells, biomolecules and tissue. AFM viscoelasticity measurement is performed by applying a small indentation to the tissue surface while recording the deflection of the cantilever [25], [26]. There is a high risk of damaging the tissue in rheology, indentation and AFM because of the application of external forces. Although MRE is a non-invasive method, a bulky expensive unit is required for this system. Ultrasound-based techniques are non-invasive, and relatively widespread with clinical applications for liver fibrosis and breast cancer providing whole-body imaging depth, but suffer from low resolution around 500 µm [27].

Photoacoustic (PA) imaging is a rapidly expanding technique that has emerged in the past decade, enabling imaging of deep tissues with high resolution [28]. This method relies on laser excitation, usually pulsed laser, which triggers a thermoelastic expansion of tissue, producing high-frequency ultrasound waves that can be detected by ultrasound transducers [29], [30] or optical methods such as interferometry [31], [32]. PA imaging (PAI) has numerous applications, ranging from small organelle to whole organ imaging [33], [34]. PA microscopy (PAM) can provide high-resolution images, offering insights into cellular morphology [35], functional status [36], [37], [38], and molecular composition [39]. PA tomography can be used to generate 3D images of deep tissues, up to several centimeters deep, by employing a wide laser beam with an array of transducers for detection [40]. PA endoscopy has also been developed for imaging internal organs such as the gastrointestinal tract [41], [42]. Given that conventional PAI is based on optical absorption, it has been demonstrated that mechanical properties, such as viscoelasticity, can also be measured using this technique [43]. The viscosity-elasticity ratio can be determined based on the phase delay between the PA signal and the laser excitation signal in the photoacoustic viscoelasticity (PAVE) system in which laser modulation is mostly in the range of kHz. PAVE has been employed for the detection of liver diseases such as hepatitis and cirrhosis [44], [45], distinguishing tumors and surrounding tissue [43], [46], identification of atherosclerosis [47], [48], [49] and esophageal disease [50], mapping mechanocellular properties [51], [52] and measuring mechanical parameters of gray and white matter of mouse brain [53]. As mentioned above, various techniques exist for tissue differentiation based on the viscoelasticity with their profits and applications, but the PAVE offers a non-destructive method same as MRE and USE. The most important advantage of the PAVE technique compared to the ultrasound or MRE methods is higher spatial resolution (around 10 times) with the cost of a lower imaging depth (a few cm). Moreover, those systems are large and expensive [27].

In the present study, the viscoelasticity of various brain areas including the cerebellum and prefrontal cortex of 5 control rats and 5 autistic-like ones has been measured by the proposed PAVE system as a reliable, low-cost, non-invasive method. Moreover, in order to make the deductions strong, the percentage of the water content of these brain regions has been investigated in 4 control rats as well as 4 rats with autism.

## 2 Methods

### 2.1 Animals

A total of 18 male offspring of Wistar rats were used for water content assessment and PAVE studies. Animals were kept at the room temperature of 23 ± 2˚C and a 12:12 h light/dark cycle with food and water ad libitum. In the present study, two groups of control and maternal VPA-treated group, referred to as autistic-like offspring, were employed and two sets of experiments were performed. The first set was carried out to assess the alterations in the viscoelasticity and the second set was done to determine the water content of the cerebellum and prefrontal cortex. Experimental protocols and animal care were done according to the guidelines approved by the Ethics Committee of Shahid Beheshti University of Medical Sciences in line with the NIH Guide for the Care and Use of Laboratory Animals (IR.SBMU.MSP.REC.1397.335).

**Fig. 1:**
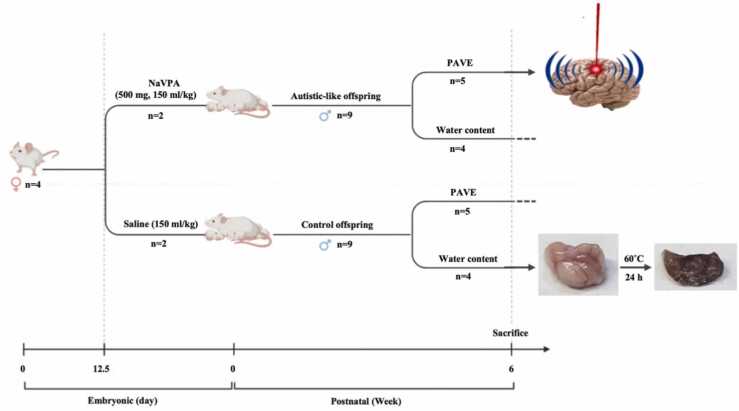
Schematic diagram of the experiments.

To induce autistic-like behavior in rats, 4 female rats (200–250 g) were mated, and pregnancy was determined by the presence of spermatozoa in vaginal smears. On the embryonic day 12.5, dams received a single intraperitoneal injection of Sodium Valproate (NaVPA, 500 mg/kg, 150 ml/kg) or saline (150 ml/kg) [54], [55], [56], [57], [58]. After weaning, offspring were separated from their dams and housed in standard cages. The 6-week male offspring were divided into separate groups for the studies (Fig. 1).

### 2.2 Principle of PAVE

**Fig. 2:**
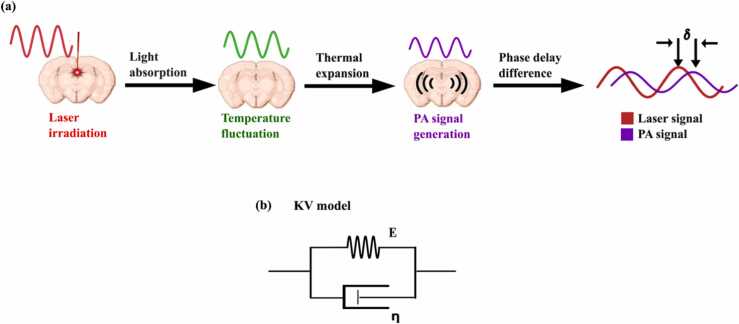
(a) Working principle of PAVE. (b) Kelvin-Voigt model for viscoelasticity.

The working principle of PAVE system is depicted in Fig. 2(a). An intensity-modulated laser beam with a sinusoidal intensity with the form of $I={0.5I}_{0}(1+\cos\omega t)$ is required for the sample irradiation. ω and $I_{0}$ represent the modulation frequency and time-average light intensity, respectively. Absorption of light by the tissue, changes the temperature of the tissue in a periodic form and thermal stress will be generated accordingly. This sinusoidal thermal stress leads to the production of periodic strain with the exact frequency as the light modulation but with a phase lag related to the viscoelastic damping effect of the tissue [59]. By employing the Kelvin-Voigt (KV) model for viscoelasticity which is a parallel connection of a spring and a damper as shown in Fig. 2(b), stress (σ)-strain (ε) relation can be calculated based on the Eq. 1 in which E and η are Young’s modulus and coefficient of viscosity accordingly [60].

(1) $$\sigma \left(t\right)=E\varepsilon \left(t\right)+\eta \dot{\varepsilon }(t)$$

After taking the Fourier transform of Eq. 1, the phase lag δ between the stress and strain or laser excitation and the generated PA signal can be found as expressed in equation 2.

(2) δ=arctan(ηω/E)

Therefore, the ratio of viscosity to elasticity has a direct relation to the amount of the phase delay between the laser and PA signal and can be used for comparing the viscoelasticity properties of different samples.

### 2.3 PAVE experimental setup

**Fig. 3:**
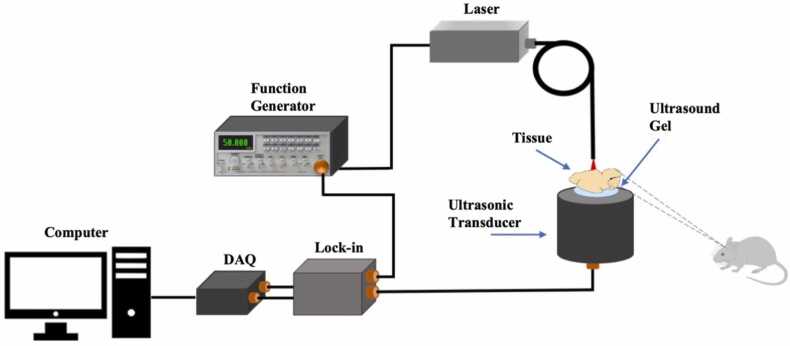
Schematic diagram of the PAVE system.

Fig. 3 illustrates the employed system for measuring viscoelasticity based on the PA effect. A fibre-coupled continuous wave laser with a wavelength of 808 nm, maximum output power of 1 W and modulated with 50 kHz frequency was chosen as the excitation source (the laser spot diameter was 0.8 mm ensuring the ANSI limit for the medical applications). The sample was placed on a thin layer of ultrasound gel working as the coupling media on top of the ultrasound transducer (UT). Both the produced PA wave, collected by a 50 kHz UT (DYW-50 kHz, Dayu Electric) with a 63 mm diameter and 10% fractional bandwidth, and the laser excitation signal are fed to a homemade lock-in amplifier for measuring the phase delay between these two signals. Finally, the signals of both channels of the lock-in amplifier were digitized by a DAQ card (USB-4716 Advantech) and transferred to the computer for further processing. The MATLAB platform was employed for controlling the system, generating a driving signal for the laser and further processing the raw data. Based on the ultra-low level of the PA signal and with the reference of its periodicity, lock-in amplification has been used. In this regard, two-channel homemade lock-in amplifier was used to measure both the amplitude and the phase of the generated signal (with the reference to the excitation). For this purpose, the PA signal and the reference signals were multiplied and then filtered to find the amplitude and the phase. In contrast to the most of previously reported PAVE systems, the primary distinction lies in the modulation method of the excitation laser. We have modulated the laser beam electronically, offering compactness, cost-effectiveness, versatile modulation formats and faster modulation compared to the electro-optic and acousto-optic modulators.

### 2.4 Phantom and sample preparation for PAVE

To verify the system performance, gelatin phantoms mimicking the mechanical properties of the biological tissues were prepared with two different concentrations of 10% and 20%. First, the gelatin powders in the water were stirred at room temperature followed by heating them in a water bath for complete dissolving and generation of a uniform solution. By pouring the solution into the molds and waiting for a few hours, the phantoms were ready for the viscoelasticity tests. In addition, to check the system capability, the liver, fat and muscle of a chicken were considered as the biological samples and their viscoelasticity was measured.

In order to assess the mechanical properties of the brain tissues in normal and autism-like conditions, rats were sacrificed following anesthesia by 100 mg/kg ketamine and 10 mg/kg xylazine, and then brains were removed, and 3 mm-thick slices of the cerebellum and prefrontal cortex were prepared. To slow down the tissue degradation and prevent tissue dehydration, acute slices were submerged in carbogenated (95% O_2_ / 5% CO_2_) artificial cerebrospinal fluid composed of (in mM): 125 NaCl, 2.5 KCl, 1.5 CaCl_2_, 1.25 NaH_2_PO_4_, 25 NaHCO_3_ and 10 D-glucose.

### 2.5 Measurement of the brain water content

The wet/dry weight technique was used to calculate the water content of the brain. After sacrifice, the brains were carefully removed from the skull. Then, 100 mg tissue of the cerebellum and prefrontal cortex were incubated for 24 h in a 60 °C oven and the initial and final weights were measured. Water content of various rat brain regions samples was expressed according to Eq. 3 [61]:

(3) Water percentage (%) = [(wet weight – dry weight) / wet weight] × 100

### 2.6 Statistical analysis

The results, depicting water content and PA phase delay, were presented as the mean±SEM (standard error of the mean). Analysis was conducted using unpaired t-test, employing GraphPad Prism software. Furthermore, differences with a significance level of p < 0.05 were considered statistically significant.

## 3 Results

### 3.1 PAVE of phantoms

**Fig. 4:**
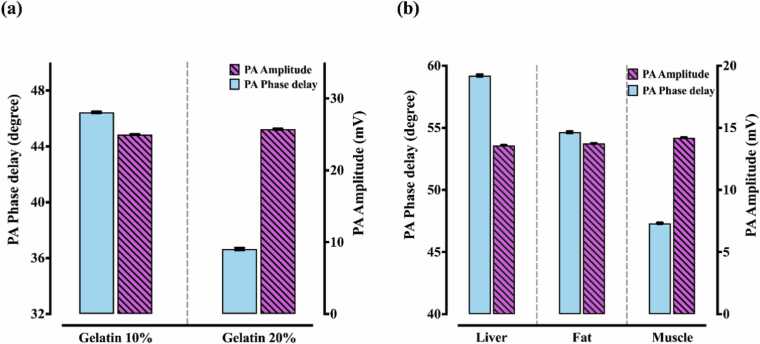
Bar chart of the PA phase delay and amplitude for (a) gelatin phantoms. (b) chicken tissues.

Viscoelasticity of the gelatin-water mixtures with 10% and 20% concentrations were measured by the proposed PAVE system and the results are presented in Fig. 4(a). The blue bars show that by doubling the concentration of gelatin samples, their PA phase delay dropped significantly from 46.44 ± 0.15 to 36.65 ± 0.23 degrees (a reduction of 22.8%) while the PA amplitude of the measured signals illustrated as purple bars represent a slight variation (24.97 ± 0.07 mV for the sample with 10% concentration vs 25.73 ± 0.07 mV for 20% concentration gelatin). Fig. 4(b) shows the evaluation of the PA phase delay (blue bars) and PA amplitude (purple bars) for the liver, fat and chicken muscle as biological tissues. It can be seen that although the PA phase delay is a good parameter for distinguishing these tissues, the variation of PA amplitude was small and remained at the same level approximately (13.6 ± 0.02 mV for liver, 13.7 ± 0.02 for fat, and 14.2 ± 0.01 for muscle). The liver stands on the first rank with the highest phase delay of 59.22 ± 0.08 degrees, followed by the fat with 54.65 ± 0.07 degrees in the second place. The minimum phase delay is related to the muscle as the stiffest tissue with 47.32 ± 0.05 degrees as expected.

### 3.2 PAVE of the mouse brain

**Fig. 5:**
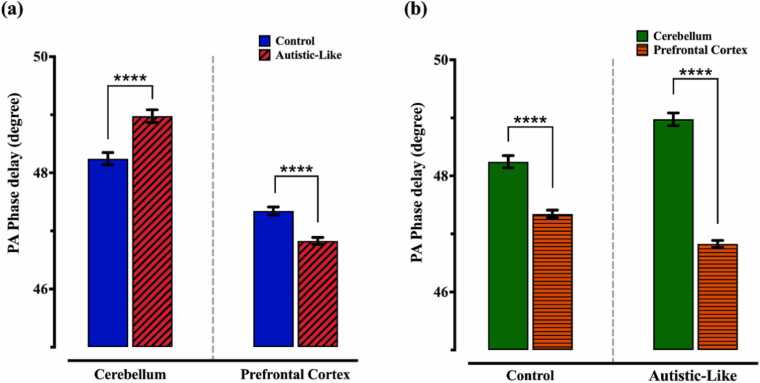
(a) PA phase delay of the cerebellum and prefrontal cortex in control and autistic-like groups. (b) PA phase delay of different regions of the control and autistic-like samples.

To assess the possible impacts of autism on the mechanical properties of different regions of the brain, PA phase delay of the cerebellum and prefrontal cortex for control and autistic-like rats were measured and the results are depicted in Fig. 5(a). The phase delay of the cerebellum for the autistic-like group is noticeably higher than the value for the control group (48.98 ± 0.11 degrees vs 48.24 ± 0.11 degrees). However, for the prefrontal cortex, the phase delay of the rats with autism is lower than the measured value for the control samples (46.83 ± 0.06 vs 47.35 ± 0.07).

To explore the viscoelasticity properties of the various regions of the brain, mean values of the PA phase delay of the cerebellum and prefrontal cortex were measured for control and autistic-like groups (Fig. 5(b)). The results express that in both groups, the cerebellum has a higher viscoelasticity ratio than the prefrontal cortex, but the difference is not the same. Cerebellum shows a higher phase delay (48.24 ± 0.11 degrees) compared to the prefrontal cortex (47.35 ± 0.07 degrees) in control rats. While the trend is the same for rats with autism, the phase delay of the prefrontal cortex is markedly lower than the value for the cerebellum (46.83 ± 0.06 degrees vs 48.98 ± 0.11 degrees). In other words, the percentage of the variation of the phase delay from the cerebellum to the prefrontal cortex increased from 1.87% to 4.39%, respectively.

### 3.3 Mouse brain water content

**Fig. 6:**
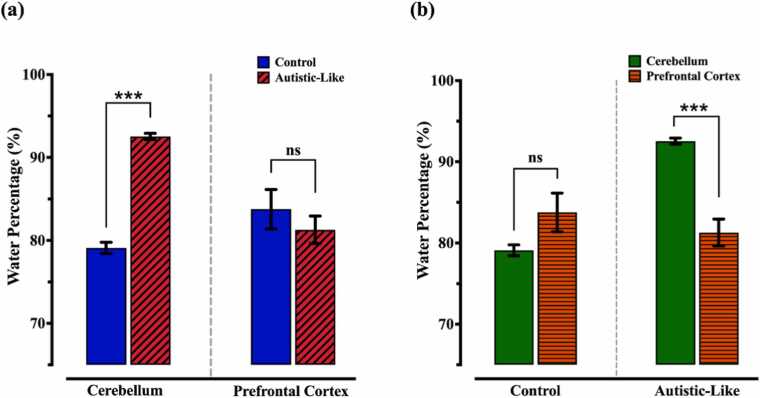
(a) Percentage of water in the prefrontal cortex (PFC) and cerebellum through the difference in wet/dry. (b) Percentage of water of various rat brain regions in the control and autistic-like groups.

Prenatal exposure to the VPA led to a significant elevation in the water percentage in the 100 mg of the cerebellum (92.54 ± 0.38 vs. 79.1 ± 0.67, p˂0.001) compared to the control. However, for a 100 mg sample of the prefrontal cortex, the water percentage did not change considerably (81.28 ± 1.65 vs. 83.77 ± 2.37, p = 0.4228) (Fig. 6(a)). Next, to investigate whether the water content is different between these two brain areas, the water percentage of the cerebellum and prefrontal cortex within rats of the same group were compared (Fig. 6(b)). In control rats, the prefrontal cortex had a slightly higher water percentage than the cerebellum, with mean values of 83.77 ± 2.37 in the prefrontal cortex vs. 79.1 ± 0.67 in the cerebellum. However, statistical analysis revealed that it was not significant (p > 0.05).

## 4 Discussion

### 4.1 Verification of the system performance

To evaluate the system performance, the PA phase delay of gelatin samples with 10% and 20% concentrations were measured and compared to similar studies in the literature. According to the values provided by Zhao et al., by increasing the gelatin concentration from 4% to 7%, the percentage of change in PA phase delay was around 22% [62]; the results of our setup showed a 21.1% change in PA phase delay by doubling the gelatin concentration, meaning a good agreement to the reported values. Furthermore, the PA phase delay of a chicken's liver, fat and muscle was assessed and a change of 12.9% was measured between the PA phase delay of the fat and muscle. Gao et al., employed the PAVE system to investigate the viscoelasticity of the liver, fat and muscle of a pig [63], the trend of the PA phase delay of these tissues was similar to our findings. Moreover, the pig’s fat had an 8.2% higher phase delay in comparison to the muscle of the pig. Given that the tissue composition of chicken and pig as well as the sample preparation in these two studies might differ, this small difference seems reasonable.

### 4.2 Water content of brain tissue

The areas that are crucial brain regions implicated in ASD pathogenesis include the prefrontal cortex, amygdala, hippocampus, and essentially the cerebellum. Here, we selected the prefrontal cortex and cerebellum brain regions that are involved in motor learning [64], which is disrupted in ASD [65]. We demonstrated that prenatal exposure to VPA significantly increased the cerebellar water content in VPA-induced autistic-like offspring. In agreement with our results, Deckmann et al. revealed a significant increase in the whole brain water percentage of the VPA animal model of autism [66]. Furthermore, Kumar et al. have recently shown a huge cerebellar permeability [67] and increased water content in the cerebellum [68] of rats treated with prenatal VPA.

As a rule of thumb, there is a direct relationship between water content and tissue viscoelasticity[8]. Interestingly, here we provide the first evidence for the accompanying decreased elasticity by the increment of water content in the cerebellum of the autistic-like model.

In the other respect, previous studies demonstrated that white matter stiffness and myelin content exhibit a strong correlation [7], and the brain water level changes during the myelination process. As the brain matures, increasing myelination shows a concomitance decrease in brain water content, also shown in the cerebellum [69], [70]. Moreover, as mentioned previously, there is an interlink between the mechanical properties of the brain, such as viscoelasticity with brain composition [7] and gray and white matter (myelinated axons) characteristics [6]. However, there is a shred of evidence demonstrating delayed myelination in the animal models of ASD during development [71]. It can be concluded as a suggestion that the possible delayed myelination in autism would have resulted in the higher water content in the cerebellum of autistic-like offspring.

Since the higher water content reflects the presence of higher liquid volume in the brain of autistic-like animals, it would be proposed that brain oedema in ASD possibly is a factor leading to higher brain volume regions in autistic patients. In keeping with this claim, MRI findings in ASD individuals have well described the volumetric differences in the total brain [72], [73] and numerous subcortical structures, especially the cerebellum [7], [8], as compared to typically developing children. In accordance, greater brain volume [73] and enlarged cerebellar volume have been detected in ASD patients.

We also examined the content of water in the PFC region. Although the changes were not significant, a reduction in water percentage appeared in the PFC of offspring affected by prenatal exposure to VPA compared to the control group. Bolduc et al. and Limperopoulos et al. have shown that prenatal cerebellar malformation led to a relative reduction in the volume of the prefrontal cortex at age two, possibly the age onset of ASD [74], [75], [76]. According to the critical association of the cerebellum and prefrontal cortex and the structural and functional connection between them, these findings support the importance of cerebellum and prefrontal cortex mechanical abnormalities in autism development.

### 4.3 PA viscoelasticity of the brain tissue

As the tissue structure and constituent can mainly determine its mechanical properties, such as viscoelasticity, assessing the water content can help the results interpretation. Mchedlishvili et al. investigated the effect of oedema development in the rabbit's brain tissue, in which they showed that by increasing the water content of the tissue, as a marker of oedema formation, tissue compliance elevated considerably [77], meaning a decline in the tissue elastic modulus and therefore growth in the viscosity-to-elasticity coefficient. Benjamin et al. studied the effect of age on the mechanical properties of various brain regions, including the hippocampus and cortex. They reported that there is a negative correlation between the water content and stiffness (elastic modulus) [78] or a direct relationship between water content and viscoelasticity. According to the Fig. 6(a), the water content of the cerebellum in the autistic rats is significantly higher than the control ones, which confirms the larger phase delay for the rats with autism (Fig. 5(a)). The water content of the prefrontal cortex and cerebellum in autistic-like samples (Fig. 6(b)) determines that prefrontal cortex is remarkably stiffer than the cerebellum, which justifies the lower viscosity-to-elasticity coefficient for prefrontal cortex (Fig. 5(b)). The water content of the prefrontal cortex in control and autistic-like groups, as well as the prefrontal cortex and cerebellum in control tissues, are not statistically different; while there is a noticeable change in their viscoelasticity properties. A possible explanation is that the differences in cellular composition, water, protein and lipid contents may also lead to the variation in mechanical properties, particularly the viscosity-elasticity ratio [79]. MacManus et al. evaluated the shear modulus of various brain regions, including the cerebellum and cortex of different species such as mouse, rat and pig for adolescents and young adults with the indentation method. They observed that the shear modulus of the rat’s cortex was significantly higher than the value for the cerebellum at all ages [79]. Considering the structural similarity between the prefrontal cortex and other cortices, the findings of MacManus et al. can be extended to the shear modulus of the prefrontal cortex and cerebellum. On the other hand, Young’s modulus can be calculated from Eq. 4, which represents the relation between shear modulus and Young’s modulus [80].

(4) E=2(ν+1)G

where G and ν are shear modulus and Poisson’s ratio, respectively. As the tissue can be considered as an incompressible medium due to the high water content, the Poisson’s ratio is around 0.5 and the elastic modulus can be approximated by the three-fold of shear modulus. Therefore, it is expected that in control samples, Young’s modulus for the cerebellum would be lower than the prefrontal cortex. Fig. 5(b) shows that the cerebellum has a higher phase delay, meaning viscoelasticity, compared to the prefrontal cortex. These results are in good correspondence with the literature since there is an inverse relation between Young’s modulus and viscosity-to-elasticity coefficient.

## 5 Conclusions

To the best of our knowledge, there has been no study investigating the impact of autism induction on the viscoelasticity properties of different brain regions measured by PAVE system. In the present study, the application of PAVE for acquiring the viscoelasticity of various brain regions including the cerebellum and prefrontal cortex for control and autistic-like conditions has been demonstrated. The results of our study reveal that the cerebellum in control samples is noticeably stiffer than in autistic tissues. On the contrary, the viscosity-to-elasticity coefficient of the prefrontal cortex in control groups was higher than the measured value for the rats with autism. In addition, a comparison between the viscoelasticity of the brain regions showed that the prefrontal cortex had a lower visco-elasticity ratio than the cerebellum for both control and autism samples. While the mechanical properties of the tissues are completely dependent on their composition and structure, the percentage of water content was calculated for different conditions to support the deduction. There are some requirements which should be considered for sample preparation that might limit the applications of the PAVE system. The samples should have the same thickness and their surface must be flat to avoid the undesired phase deviation due to the thickness difference or uneven surfaces. Our study’s findings present a novel avenue for investigating the autism using a trustworthy, economical, and non-intrusive methodology based on mechanical characteristics. In light of the significance of mechanical properties of the brain tissues in neuroscience research, our suggested approach for quantifying brain tissue viscoelasticity holds the potential for effective utilization.

## Declaration of Competing Interest

The authors declare that they have no known competing financial interests or personal relationships that could have appeared to influence the work reported in this paper.

## Data Availability

Data will be made available on request.
